# Worker Body Size Drives Cuticle Thickness and Higher Relative Intensity of the Lipid Reaction in the Leaf-Cutting Ants *Atta laevigata* and *Atta sexdens*

**DOI:** 10.1007/s13744-026-01436-0

**Published:** 2026-09-29

**Authors:** Iasmin Góes Frossard, Kênia Aparecida dos Santos Mateus, Karina Dias Amaral, Diego dos Santos Souza, Jéssica Josefa Sanches, Catarina de Lima Januario, Elane Borba Alves, José Eduardo Serrão, Ronald Zanetti

**Affiliations:** 1 Dept of Entomology, Univ Federal de Lavras, Lavras, Minas Gerais Brazil; 2 Dept of Entomology, Univ Federal de Viçosa, Viçosa, Minas Gerais Brazil

**Keywords:** Polymorphism, Subcastes, Surface-area-to-volume ratio, Water balance

## Abstract

The body size of insects influences the surface-area-to-volume ratio and can affect the thickness of the cuticle and relative intensity of the lipid reaction particularly in eusocial species such as leaf-cutting ants of the genus *Atta*, which exhibit pronounced worker polymorphism. In this study, we evaluated the relationship between body size, relative intensity of the lipid reaction, and cuticle thickness in workers of *Atta laevigata* (Smith, 1858) (Hymenoptera: Formicidae) and *Atta sexdens* (Linnaeus, 1758) (Hymenoptera: Formicidae). Small, medium, and large workers, defined according to the width of the cephalic capsule, were compared regarding relative cuticle thickness and relative intensity of the lipid reaction. Both traits varied among worker size classes. Small workers showed higher relative intensity of the lipid reaction and greater relative cuticle thickness, while medium and large workers exhibited lower values for both traits, indicating that different cuticle components respond similarly to body size, regardless of the species. Although the relative intensity of the lipid reaction was not differentiated between species, cuticle thickness was consistently greater in *A. sexdens* than in *A. laevigata* in all size classes. Overall, our findings suggest that differences in cuticular characteristics between *A. laevigata* and *A. sexdens* are not solely associated with body size and may also be related to the specific functions performed by workers within the colony.

## Introduction

The body size of insects directly influences important physiological processes, including thermoregulation, gas exchange, and water balance (Bouchebti et al. 2015; Buxton et al. 2021). Smaller insects have a higher surface-area-to-volume ratio, which proportionally increases the body area exposed to the environment (Chown et al. 2011; Kühsel et al. 2017). Consequently, they gain and lose heat more rapidly in response to ambient temperature and humidity. Elevated body temperatures accelerate metabolic activity and gas exchange rates, leading to greater water loss through the cuticle and tracheal spiracles (O'Donnell 2022). In contrast, larger insects, with a smaller surface-area-to-volume ratio (Kühsel et al. 2017), warm up more slowly, perform gas exchange less frequently, and lose proportionally less water, making them less susceptible to dehydration (Bouchebti et al. 2015; Brückner et al. 2017).

The main barrier against water loss in insects is the cuticle (Chown et al. 2011), which is composed of three distinct layers. The epicuticle, the outermost layer, is rich in lipids, particularly cuticular hydrocarbons, which form an effective barrier, reducing water loss, protecting against pathogens, and mediating intra- and interspecific communication in social insects (Howard and Blomquist 2005). Below this layer, the exocuticle and endocuticle are composed mainly of chitin and associated proteins, providing both rigidity and flexibility to the exoskeleton (Wigglesworth 1948). The amount of chitin, proteins, and lipids in the insect cuticle is directly related to body size, since larger insects require greater amounts of cuticular material to cover a proportionally larger surface area (Evans and Sanson 2005). Despite this, relatively little is known about how body size influences other cuticle attributes, such as thickness and the relative intensity of the lipid reaction, especially in eusocial species, where individual size is often associated with colony function, imposing additional selective pressures on the cuticle (Peeters et al. 2017).

Leaf-cutting ants exhibit a highly organized collective foraging system, in which worker ants explore the environment in search of plant material, cutting and transporting this material to the interior of the colony, where it will be used to cultivate the symbiotic fungus (Della Lucia 2011). The performance of the worker ants during foraging is crucial, as it directly influences the quantity and quality of the collected material and, consequently, the growth of the fungus and the maintenance of the colony (Bass and Cherrett 1995). This performance depends, among other factors, on the body size of the worker ants, which influences both the size of the load they can carry and the speed of movement, as well as determining how these individuals respond to the thermal conditions of the external environment (Kalinkat et al. 2015; Kühsel et al. 2017).

Leaf-cutting ants of the genus *Atta* constitute an interesting model for studying the interaction between body size and cuticular characteristics. In addition to the differences between reproductive and non-reproductive castes, the worker caste is subdivided into several size categories, associated with different behavioral roles and levels of environmental exposure (Hölldobler and Wilson, 1990; Peeters et al. 2017). Medium-sized (≈1.4 mm) and large (≈2.0–2.2 mm) workers are predominantly involved in foraging and nest defense, activities that expose them to adverse external conditions such as low humidity and high temperatures (Santos et al. 2022; Püffel et al. 2023). Their larger body size reduces the surface-area-to-volume ratio, making them less prone to dehydration (Kühsel et al. 2017). In contrast, smaller workers (≈0.8–1.0 mm), which primarily perform internal colony tasks, including maintaining the fungus garden, building the nest, and caring for the brood (Santos et al. 2022; Püffel et al. 2023), have a larger surface-area-to-volume ratio and are therefore more vulnerable to water loss (Kühsel et al. 2017). Although these workers are predominantly associated with intranidal activities, they may also occur on external foraging trails as hitchhikers, traveling on leaf fragments carried by larger workers (Vieira-Neto et al. 2006; Souto 2012).

In this context, this study aimed to evaluate the relationship between body size, cuticle thickness, and relative intensity of the lipid reaction in worker ants of the leaf-cutting ants *Atta laevigata* (Smith, 1858) and *Atta sexdens* (Linnaeus, 1758). Specifically, both intraspecific variation among workers of different sizes and interspecific differences were investigated. The hypothesis to be tested is that smaller workers of both species exhibit greater relative cuticle thickness and higher relative intensity of the lipid reaction than larger workers, possibly as a consequence of their greater susceptibility to water loss associated with smaller body size, due to their higher surface-area-to-volume ratio, as well as potential episodes of exposure to the external environment during indirect foraging activities. In addition, the hypothesis that *A. laevigata* exhibits greater cuticle thickness and a higher lipid reaction intensity than A. sexdens was evaluated based on differences in thermal tolerance between the species. *Atta laevigata* shows a higher upper lethal temperature than *A. sexdens* (37.5°C and 36.3°C, respectively) and a lower specific metabolic rate at 25°C, indicating physiological differences between the species that may be related to their distinct thermal tolerances (Joséa et al. 1981; Hebling Beraldo and Mendes 1982).

## Materials and methods

### Collection of ant workers

Workers from three colonies of *A. laevigata* and three colonies of *A. sexdens* were collected directly from the fungus gardens following the partial excavation of nests located in an area of shaded native vegetation, in the municipality of Viçosa, Minas Gerais state, Brazil (20°45′S, 42°52′W). Three *A. laevigata* nests and three *A. sexdens* nests were selected. Nest size was estimated based on the area of ​​the loose soil mound, calculated from measurements of the maximum length and width (Moreira et al. 2004). The sizes of *A. laevigata* nests were 21.3 m^2^, 34.0 m^2^, and 40.6 m^2^ of loose soil, whereas the *A. sexdens* nests measured 20.5 m^2^, 23.8 m^2^, and 42.4 m^2^. The study area is characterized as a Seasonal Semideciduous Montane Forest, within the Atlantic Forest domain and located in the Zona da Mata region of Minas Gerais, Brazil. The habitat consists of a fragment of submontane tropical forest, which serves as an important refuge for local biodiversity. The regional climate shows an average annual temperature between 19 and 20°C and annual precipitation ranging from 1260 to 1350 mm, with rainfall concentrated mainly between October and March.

### Size of workers

A total of 24 workers per size class were selected for each species, comprising eight workers from each of the three colonies. Thus, 24 small, medium, and large workers were analyzed per species. Workers were photographed using a camera coupled to a Zeiss Stemi 2000 C stereomicroscope. The images were used to measure head capsule width, defined as the maximum distance across the head, using ImageJ software (Baudier et al. 2015), which was adopted as the criterion for size classification (Wilson 1980). Workers with head capsule widths between 0.8 and 1.0 mm were classified as small, those between 1.4 and 1.6 mm as medium, and those between 2.0 and 2.2 mm as large, following Wilson (1980) and Santos et al. (2022).

### Relative intensity of the lipid reaction and cuticle thickness

For each species, 24 workers per size class (small, medium, and large) were selected, comprising eight workers from each of the three colonies. The same workers were photographed, their head capsule width measured, and used in the analyses of cuticle thickness and relative intensity of the lipid reaction. The pronotum was carefully removed using scissors and forceps. The pronotum is commonly used in studies of ant cuticle thickness because it represents a morphologically uniform region, allowing for reliable comparisons among individuals (Peeters et al. 2017). After dissection, the pronotum was transferred to 4% paraformaldehyde in 0.15 M sodium phosphate buffer (pH 7.2) fixative solution for 24 h. The samples were then rinsed in the same buffer, dehydrated through a graded ethanol series (70%, 80%, 90%, and 95%), and embedded in glycol methacrylate historesin (Leica Biosystems Inc.) according to the manufacturer’s instructions. Sections 2 µm thick were obtained using a Leica RM2255 motorized microtome and stained with Nile blue to visualize the lipid reaction (Bancroft and Gamble 2008). The sections were mounted, examined, and photographed using an Olympus BX60 light microscope equipped with a Q-Color digital camera. All images were obtained using a 20×, 0.40 numerical aperture objective lens under identical light intensity and exposure settings to ensure consistency among samples.

### Morphometry

Images of pronotum slices from each worker were analyzed using ImageJ software to quantify the relative intensity of the lipid reaction, following a standardized protocol based on the intensity of the Nile blue histochemical reaction. Briefly, images of the pronotum cuticle were converted to 32-bit grayscale and inverted to negative. In each image, five non-overlapping areas with standardized dimensions (64 × 64 pixels) were selected from anatomically corresponding areas of the pronotum, ensuring that the relative intensity of the lipid reaction was quantified from the same cuticular region in all individuals. The mean gray values obtained from these regions were used to calculate the average Nile blue staining intensity for each individual, serving as a semiquantitative proxy of relative intensity of the lipid reaction (Urstadt 2022).

**Fig. 1:**
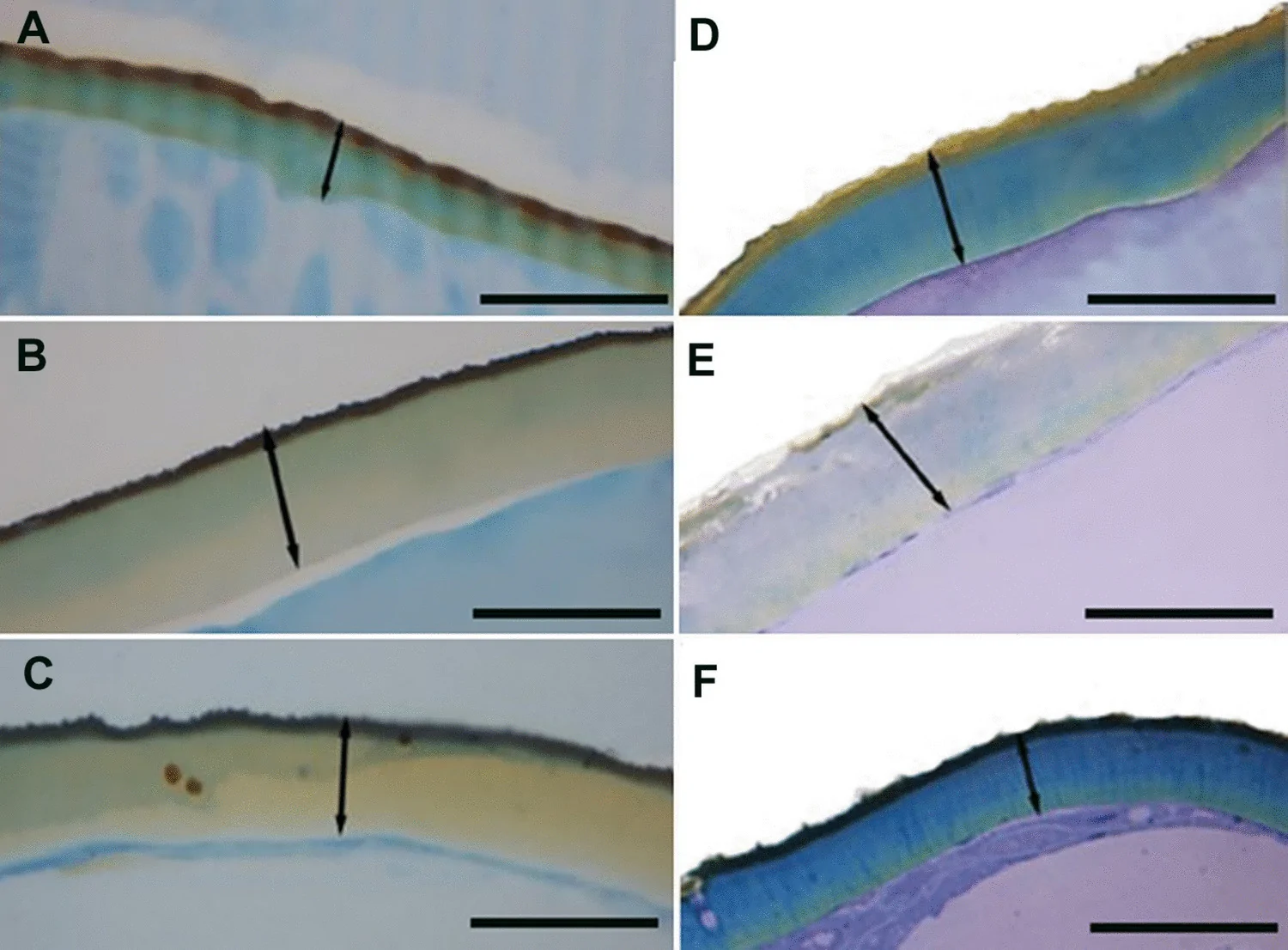
Transverse histological sections of the pronotum of small, medium, and large workers of *Atta laevigata* **A**–**C** and *Atta sexdens* **D**–**F**. **A**, **D** Large workers; **B**, **E** medium workers; **C**, **F** small workers. The images indicate the region used for measuring cuticle thickness and relative intensity of the lipid reaction. Cuticle thickness was determined by drawing a line perpendicular to the cuticular surface, from the external edge to the base of the cuticle. Relative intensity of the lipid reaction was quantified in standardized areas of the same histological section. Scale bars = 50 µm

Cuticle thickness was measured in the same images by drawing a line perpendicular to the cuticular surface, from the outer edge to the basal boundary of the cuticle (Fig. 1). To allow comparisons among workers of different sizes, both cuticle thickness and relative intensity of the lipid reaction were standardized by body size. Values were divided by head capsule width (Whyte et al. 2023), used as a proxy for body length (Weiser and Kaspari 2006). This procedure yielded estimates of relative cuticle thickness (µm of cuticle per mm of head capsule) and relative intensity of the lipid reaction (arbitrary units per mm of head capsule, AU mm⁻^1^).

### Statistical analyses

To evaluate the effects of worker size and species on relative intensity of the lipid reaction and relative cuticle thickness, the data were analyzed using linear mixed-effects models (LMMs). In the models, worker size and species were treated as fixed effects, while nest identity was included as a random effect to account for the hierarchical structure of the data and avoid pseudorepetition. Models were fitted using the *lme4* package, and the significance of fixed effects was assessed using Satterthwaite’s approximation for degrees of freedom implemented in the *lmerTest* package. Model assumptions were evaluated through the analysis of simulated residuals using the *DHARMa* package, ensuring compliance with assumptions of normality and homoscedasticity. This modeling framework allows the biological effects of interest to be isolated from environmental or genetic variation shared within colonies. All statistical analyses were conducted in R version 4.5.0 (R Core Team 2025), adopting a significance level of 0.05.

## Results

**Fig. 2:**
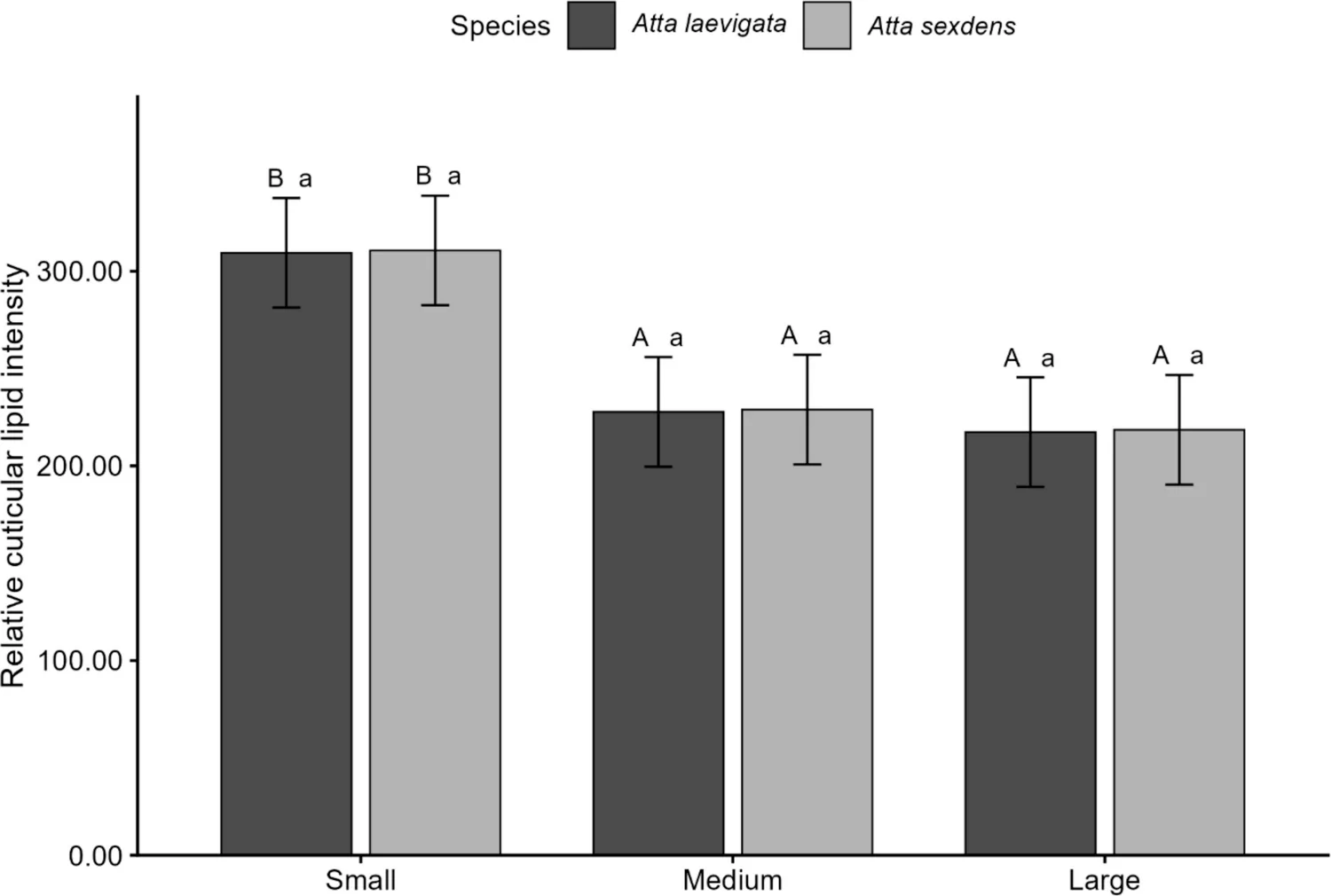
The relative intensity of the lipid reaction (values ​​proportional to head capsule size) of *Atta laevigata* (dark gray) and *Atta sexdens* (light gray) workers in three size classes (small, medium, and large). Bars represent means ± standard errors. Small letters indicate significant differences between sizes within each species; capital letters indicate differences between species within the same size (multiple comparison test, *p* < 0.05)

The relative intensity of the lipid reaction estimated from Nile blue staining intensity did not differ between species (F₁,₁₃₈ = 0.0157, *p* = 0.90), but varied between worker size classes in both species (F₂,₁₃₈ = 38.17, *p* = 6.41 × 10⁻^14^). Small workers of both species exhibited a higher relative intensity of the lipid reaction than medium and large workers, which did not differ from each other (Fig. 2).

**Fig. 3:**
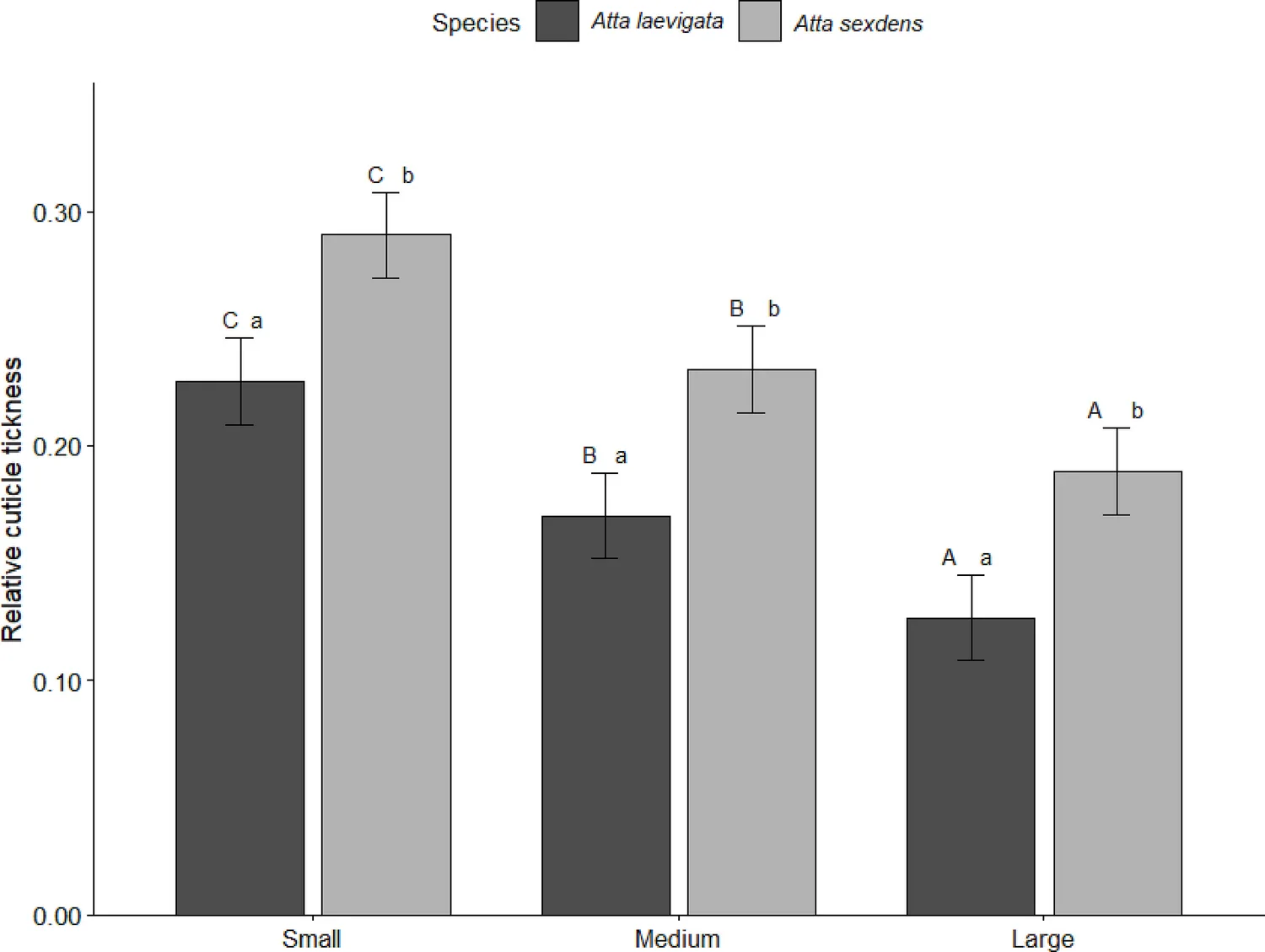
Relative cuticle thickness (values proportional to head capsule size) of *Atta laevigata* and *Atta sexdens* workers in three size classes (small, medium, and large). The bars represent the means ± standard errors. Capital letters indicate significant differences between sizes within each species; small letters indicate differences between species within the same size (multiple comparison test, *p* < 0.05)

The relative cuticle thickness varied between species (*F*₁,₁₃₈ = 85.363, *p* < 2.2 × 10⁻^1^⁶) and between worker size classes (*F*₂,₁₃₈ = 74.857, *p* < 2.2 × 10⁻^1^⁶). In all size classes, *Atta laevigata* showed lower relative cuticle thickness values ​​than *Atta sexdens* (*p* < 0.0001). Furthermore, in both species, the relative cuticle thickness decreased with worker size, with smaller workers showing the highest values, followed by medium and large workers, all differing significantly from each other (Fig. 3).

## Discussion

Worker ants of both species exhibited higher relative intensity of the lipid reaction and greater relative cuticle thickness in smaller workers. This pattern may be associated with their lower thermal inertia and higher susceptibility to water loss (Kühsel et al. 2017). It should be noted that cuticle thickness values are relative (standardized by head capsule width) and thus reflect size-adjusted investment rather than absolute thickness. The relative intensity of the lipid reaction may be associated with reduced cuticle permeability, thereby mitigating the greater water loss expected in smaller body sizes (Gibbs and Rajpurohit 2010; O’Donnell 2022). Similarly, a thicker cuticle in smaller workers could be associated with structural differences in the cuticle, which could influence water diffusion through the exoskeleton (Hadley 1994). Smaller workers of *Atta cephalotes* (minims, typically with head widths of approximately 0.8–1.6 mm) have been shown to possess relatively thicker cuticles than larger ones (Peeters et al. 2017; Constantino et al. 2021), supporting the hypothesis that cuticular traits vary in relation to body size in leaf-cutter ants.

Furthermore, greater relative cuticle thickness may be associated with differences in body mass and possibly related to variations in the mechanical costs of locomotion and cargo transport. One study demonstrated that the mechanical work required for movement and load carrying increases with body mass in the harvester ant *Messor barbarus* (Merienne et al. 2021). These costs may be particularly relevant for medium- and large-sized workers, which routinely perform external tasks such as foraging and material transport (Santos et al. 2022; Püffel et al. 2023). On the other hand, cuticle thickness does not always follow this pattern across different ant species. In *Dorylus orientalis* (Dorylinae), *Pheidole noda* (Myrmicinae), and *Cataglyphis bombycina* (Formicinae), larger workers possess thicker cuticles (Peeters et al. 2017). Notably, *C. bombycina* forages during the hottest periods of the day in the Sahara Desert, and its larger workers, which are more active under these extreme conditions, exhibit higher thermal tolerance, allowing prolonged foraging activity (Willot et al. 2018). Due to the greater surface-area-to-volume ratio associated with smaller insects (Kühsel et al. 2017), smaller *Atta* spp. workers may be more vulnerable to water loss when acting as hitchhikers during foraging activities (Vieira-Neto et al. 2006; Souto 2012), although these workers are typically associated with tasks inside the nest.

This increased susceptibility could favor a greater relative intensity of the lipid reaction and thicker cuticles, which could act as potential barriers against desiccation. In contrast, larger workers are primarily associated with foraging and nest defense, activities that expose them to adverse environmental conditions such as low humidity and high temperatures (Helanterä and Ratnieks 2008; Püffel et al. 2023). However, their smaller surface-area-to-volume ratio may reduce their susceptibility to desiccation (Kühsel et al. 2017), possibly allowing for lower investment in cuticle thickness and relative intensity of the lipid reaction in workers of both studied species.

Regarding interspecific differences, the evaluated hypothesis was that *A. laevigata* exhibits greater cuticle thickness and a higher relative intensity of the lipid reaction across all worker size classes compared to *A. sexdens*. This hypothesis is supported by differences in thermal tolerance between the species. *Atta laevigata* shows a maximum lethal temperature of 37.5°C, higher than that of *A. sexdens* (36.3°C). Taken together, these characteristics suggest that *A. laevigata* has greater tolerance to heat exposure, making it plausible that this species may exhibit a higher investment in cuticular structures as protection against a potentially increased risk of desiccation. However, the results obtained do not support this hypothesis, as *A. sexdens* exhibits greater cuticle thickness across all evaluated size classes, and no differences were found between species in relative intensity of the lipid reaction. These findings indicate that cuticle thickness and relative intensity of the lipid reaction do not, on their own, explain the interspecific differences expected based on the higher thermal tolerance attributed to *A. laevigata*. In this context, it is possible to suggest that the higher tolerance of this species may be associated with other cuticular traits not assessed in the present study, such as ventilation patterns (Lighton 1996, 1992; Perl and Niven 2018), epicuticular lipid composition (Hood and Tschinkel, 1990), cuticular hydrocarbon profiles (Gibbs and Rajpurohit 2010; Gibbs and Johnson 2004), pilosity, sculpture (Buxton et al. 2021), or levels of abrasion (Johnson et al. 2011), all of which may contribute to protection against water loss and other potential environmental pressures.

A plausible explanation for the greater cuticle thickness and the absence of differences in relative intensity of the lipid reaction may be related to differences in colony size between the two species tested. Larger colonies may exhibit a lower relative investment in cuticular structures relative to body size, possibly associated with the metabolic costs of their production (Peeters et al. 2017; Matte et al. 2025). Although nests of similar sizes were used in the present study, the interspecific differences observed, such as the greater cuticle thickness in *A. sexdens*, may indicate that this species forms larger colonies than *A. laevigata*. However, this hypothesis remains speculative, as colony size was not directly measured in this study, and further investigation is needed to test it.

The contrasting cuticular patterns observed among worker size classes and between the two species in the present study, as well as in other ant species (Peeters et al. 2017; Willot et al. 2018; Birkenfeld et al. 2024), are consistent with the hypothesis that cuticular protection in ants is not determined by a single morphological trait. Instead, these patterns may reflect interactions among cuticle thickness, lipid composition, and body size, which may contribute to adaptation to diverse environmental challenges faced by different species.

## Conclusion

Small workers present greater relative cuticle thickness and higher relative intensity of the lipid reaction than medium and large workers, a pattern that may possibly be related to their greater susceptibility to water loss due to a higher surface-area-to-volume ratio. On the other hand, larger workers, despite being more exposed during foraging and nest defense, present lower relative cuticle thickness and relative intensity of the lipid reaction, possibly due to their lower susceptibility to desiccation, due to their lower surface-area-to-volume ratio. At the interspecific level, *A. sexdens* presented greater cuticle thickness in all size classes, possibly reflecting greater availability of resources for the synthesis of metabolically costly structures, whereas *A. laevigata* may show greater investment in other cuticular characteristics not evaluated in the present study, such as more efficient gas exchange patterns, epicuticular lipid composition, and pilosity, all of which may contribute to greater protection against water loss. These patterns suggest that cuticular traits in *Atta* spp. may be associated with physiological constraints and worker ecology, with potential implications for task allocation, tolerance to extreme environmental conditions, and resource exploitation.

## Data Availability

The data that support the findings of this study are available from the corresponding author upon reasonable request.
